# Changes in the probability of hysterectomy in the city of Mainz and Mainz-Bingen region, Germany

**DOI:** 10.1186/s12889-022-14916-w

**Published:** 2023-01-11

**Authors:** Luana F. Tanaka, Olaf Schoffer, Jochem König, Veronika Weyer-Elberich, Maria Blettner, Stefanie J. Klug

**Affiliations:** 1 Chair of Epidemiology, TUM Department of Sport and Health Sciences, Technical University of Munich, Munich, Germany; 2 Center for International Health, Ludwig Maximilian University of Munich (LMU), Munich, Germany; 3 Center of Evidence-Based Health Care, Faculty of Medicine, University Hospital Carl Gustav Carus, TU Dresden, Dresden, Germany; 4 Institute of Medical Biostatistics, Epidemiology and Informatics, University Medical Center, Johannes Gutenberg University, Mainz, Germany; 5 Institute of Biostatistics and Clinical Research, University of Münster, Münster, Germany

**Keywords:** Gynaecological surgery, Epidemiology, Missing data, Inverse-probability-weighting, Kaplan–Meier

## Abstract

**Background:**

To assess the hysterectomy probability by calendar period and age, the overall and the age-specific prevalence of hysterectomy in women aged 30–65 years.

**Methods:**

Baseline data (2005–2007) from the population-based MARZY study conducted in Mainz and Mainz-Bingen, Germany, were analysed. 6429 women aged 30–65 years were asked whether they had undergone a hysterectomy and the date and indication of the procedure. We calculated the 5-year age-specific prevalence of hysterectomy and estimated the probability of undergoing a hysterectomy combining two approaches: 1) Kaplan–Meier and 2) Inverse probability weighting (IPW). We assessed potential changes over calendar periods by simulating survival curves, having hysterectomy as the event, employing a Cox proportional hazard model.

**Results:**

Data on hysterectomy were available for 4719 women. Of these, 961 (20.4%) had undergone a hysterectomy between 1960 and 2006. The hysterectomy prevalence was highest among the 60–64 year-olds (40.7%). The IPW-corrected probability of having a hysterectomy up to the age of 65 years was 36.4%. The age-specific probability of hysterectomy increased from 0.1% (20–24 years), peaking at 45–49 years (7.8%) and declining thereafter to less than 5% among women aged 50 and older. Over time, women were hysterectomised at an increasingly older age. Most hysterectomies (86.7%) were done due to benign disease.

**Conclusions:**

A shift to older age at hysterectomy with an advancing calendar period likely reflects changes in clinical practice in Germany.

**Trial registration:**

Landesärztekammer Rheinland-Pfalz: 837.438.03 (4100).

**Supplementary Information:**

The online version contains supplementary material available at 10.1186/s12889-022-14916-w.

## Background

Hysterectomy is one of the most common gynaecological surgery in many industrialised countries [1]. In Germany, more than 100 000 procedures were performed in 2019 [2]. Hysterectomies have widespread economic consequences for health systems worldwide due to the high costs involved [3] and can significantly impact a woman's physical and mental health [4, 5].

Indications for hysterectomy include leiomyoma, bleeding disorders, genital prolapse, adenomyosis, endometriosis, as well as precancerous and cancerous lesions of the female reproductive system [6]. But with the emergence of new medicamentous management and uterine-sparing surgical procedures for some of these conditions, a shift away from hysterectomy has been reported in several countries [8–11]. Common treatments for leiomyoma include the use of gonadotropin-releasing hormone agonist, levonorgestrel-releasing intra-uterine device, uterine artery embolization, myomectomy (hysteroscopic or laparoscopic), cryomyolysis and thermo-coagulation high-frequency magnetic resonance-guided focused ultrasound surgery [7]. As for genital prolapse, various approaches to pelvic reconstructive surgery are available [12].

Previous studies in Germany have either performed cross-sectional analyses of hysterectomy, reporting the prevalence of hysterectomy at the time of the survey [13, 14] or analysed hysterectomy incidence within a limited period [15]. Therefore, these studies have not adequately described a woman's probability of undergoing a hysterectomy during her entire lifespan.

We aimed to estimate the probability of undergoing a hysterectomy up to the age of 65 and the effects of calendar period and age on the probability of undergoing a hysterectomy in Germany. Additionally, we reported the main indications for the procedure in our study population.

## Materials and methods

### Study design

The MARZY study was a population-based cohort study on cervical cancer screening (CCS) conducted between 2005 and 2012 in the city of Mainz and the surrounding rural district of Mainz-Bingen in western Germany. MARZY examined the effect of two invitational models for CCS and investigated the age and type-specific HPV prevalence.

The MARZY study design is described in detail elsewhere [16]. In brief, 6429 women aged 30- 65 years with primary residence in the study region were randomly selected from population registries and invited to participate in the study. With the invitation letter, women received a response card and were asked to indicate whether they had undergone a hysterectomy, the date it was performed and its indications. Data on hysterectomy were also collected from non-responders via questionnaire and telephone interview.

The baseline investigation was conducted in 2005- 2007. Hysterectomy was an exclusion criterion for the MARZY cohort study, considering that women who had undergone this procedure were no longer eligible for CCS. The baseline assessment was the basis of the present analysis: the MARZY Hysterectomy Study.

### Categorisation of indication for hysterectomy

The written information was extracted and coded according to the International Statistical Classification of Diseases and Related Health Problems (ICD) 10th revision, Version 2019. Up to three indications for hysterectomy were provided and all of them were coded using the online ICD-10 platform [17]. We considered the indications provided by order of appearance, except for cancer, which was classified as the main indication regardless of its order. We grouped indications as: malignant or in situ cancer or neoplasms of uncertain and unknown behaviour (C00-C97; D00-D09 and D37-D48), leiomyoma of uterus (D25), benign neoplasm of ovary and other and unspecified female genital organs (D27-D28), other female pelvic inflammatory diseases (N73), endometriosis (N80), female genital prolapse (N81), polyp of female genital tract (N84), other non-inflammatory disorders of uterus, except cervix (N85), dysplasia of cervix uteri (N87), abnormal uterine bleeding (N92-N93), pain and other conditions associated with female genital organs and menstrual cycle (N94), maternal care for known or suspected abnormality of pelvic organs (O34) and contraceptive management (Z30). Unspecific information that did not allow for any ICD-10 code assignment was coded as missing.

### Statistical methods

We calculated the age-specific prevalence of hysterectomy based on 5-year age categories, considering women who provided information on hysterectomy (*n* = 4719). The age distribution of hysterectomised women at the time of the survey and the age distribution at hysterectomy were compared to illustrate the limitation associated with measuring prevalence at a single point since prevalence estimates only capture women who have been hysterectomised and who have survived up to the date of the survey.

The probability of undergoing a hysterectomy up to age 65 was estimated using time-to-event (survival) analysis, which accounted for censoring at the interview age. First, we computed the Kaplan–Meier estimate to determine the distribution of hysterectomy-free time up to age 65. We used age as the underlying time variable and analysed the time from birth until the hysterectomy (event) for women who had been hysterectomised. Women who had not been hysterectomised were censored at the age when the information on hysterectomy was obtained. Hysterectomised women with a missing date of hysterectomy were excluded from the analysis.

To avoid an underestimation of the hysterectomy prevalence due to the exclusion of hysterectomised women who were not able to provide the hysterectomy date, we employed the inverse probability weighting (IPW) method to estimate the cumulative probability of hysterectomy at a given age (t) [18]. The IPW method thereby corrected for the missing date of hysterectomy as follows:

$${\widehat{W}}_{IPW}\left(t\right)=\frac{h}{{h}_{d}}\left(1-S\left(t\right)\right)$$

*S* (*t*): Probability of not undergoing a hysterectomy up to the age *t*

*h*_d_: Number of hysterectomies where a date was indicated

*h*: Total number of hysterectomies

The 5-year age-specific probability of hysterectomy was calculated as the difference in the cumulative probability of hysterectomy between two consecutive age groups. In addition to the Kaplan–Meier estimate, we fitted a Cox model using the counting process method, with the calendar period (1939–1979, 1980–1989, 1990–1999 and 2000–2006) as a time-dependent covariate [19, 20]. We report the results as hazard ratios and corresponding 95% CIs. To test for non-proportional hazards, Schoenfeld residuals were used. We considered *p* values < 0.05 for the correlation between crude, logarithmic and squared residuals as a violation of the proportionality assumption. From this Cox model, we estimated calendar-period specific survivor functions. These functions can be assumed as simulated survival curves to examine the effect of calendar period and age on the probability of undergoing a hysterectomy.

We reported the frequency of hysterectomy indications and 95% confidence intervals (95% CI) obtained using the Clopper Pearson method. Indications were additionally presented by age at hysterectomy (15- 49 years; 50–65 years), calendar period (1939–1979, 1980–1989, 1990–1999 and 2000–2006) and age. The calendar period starts in 1939 (the earliest birth year among study participants) based on the assumption that women in the study were at risk of undergoing a hysterectomy since birth. The data were analysed using SAS (Version 9.3, Cary, North Carolina, USA) and R (version 4.1.0).

## Results

**Fig. 1:**
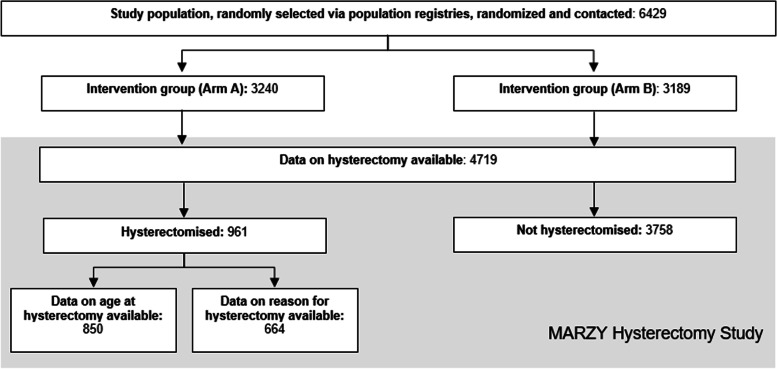
Recruitment of the MARZY Study and selection of the hysterectomised women for the MARZY Hysterectomy Study

Of the 6429 women contacted at baseline, 4719 (73.4%) provided information on whether they had undergone a hysterectomy. Of these, 961 women (20.4%) had been hysterectomised (Fig. 1). The majority of women were residents of the rural Mainz-Bingen region (57.6%) and 42.4% lived in the city of Mainz (Additional file 1). A total of 850 women (88.4%) provided information on the date when the hysterectomy was performed. All hysterectomies reported in the study population were performed between 1958 and 2006.

### Prevalence of hysterectomy in the study population

The prevalence of hysterectomy increased with age: 1.2% of women aged 30–34 years were hysterectomised compared to 40.7% of the 60–64 year-olds (Table 1). When comparing the age distribution of hysterectomised women at the time of the survey with the distribution of age at hysterectomy, the first peaked at 60–64 years (32.9%), while the latter peaked at 45–49 years (27.1%) (Table 1).

**Table 1:** Age-specific prevalence of hysterectomy (*N* = 4719), age distribution of hysterectomised women at the time of the survey (*N* = 961) and distribution of age at hysterectomy (*n* = 850), MARZY Study

Age (years)	Age distribution of study population	Age-specific prevalence of hysterectomy	Age distribution of hysterectomised women at the time of the survey	Distribution of age at hysterectomy
	**N (%)**	**(%)**	**N (%)**	**N (%)**
15–19	-	-	-	2 (0.2)
20–24	-	-	-	4 (0.5)
25–29	-	-	-	25 (2.9)
30–34	326 (6.9)	1.2	4 (0.4)	89 (10.5)
35–39	604 (12.8)	3.0	18 (1.9)	169 (19.9)
40–44	709 (15.0)	8.0	57 (5.9)	159 (18.7)
45–49	679 (14.4)	16.2	110 (11.4)	230 (27.1)
50–54	596 (12.6)	21.5	128 (13.3)	92 (10.8)
55–59	647 (13.7)	34.0	220 (22.9)	59 (6.9)
60–64	776 (16.4)	40.7	316 (32.9)	19 (2.2)
65	382 (8.1)	28.3	108 (11.2)	2 (0.2)
**Total**	**4719 (100.0)**	**20.4**	**961 (100.0)**	**850 (100.0)**

Data were only available for women between the ages of 30 and 65

### Age-specific probability of undergoing a hysterectomy

Based on the cohort excluding hysterectomised women with missing date of hysterectomy, an estimated 67.8% (95% CI 65.4—70.1%) of women would reach the age of 65 years without having undergone a hysterectomy. The IPW-corrected cumulative probability of undergoing a hysterectomy by age 65, representing the cohort without the above-mentioned exclusion, was 36.4%. The IPW-corrected age-specific probability of hysterectomy steadily increased up to the age of 45 to 49 years (7.8%) and then decreased thereafter, reaching 1.0% at age 65 (Table 2).

**Table 2:** Age-specific probability of hysterectomy (*n* = 4719), MARZY Hysterectomy Study

Age (years)	Kaplan–Meier estimation regarding hysterectomy-free time (95% CI)^a^	Cumulative probability^*^ of hysterectomy (IPW-corrected)	Age-specific probability^*^ of hysterectomy	Age-specific probability^*^ of hysterectomy (IPW-corrected)
0–14	100.0 (100.0; 100.0)	0.0	-	-
15–19	100.0 (99.8;100.0)	0.0	0.0	0.0
20–24	99.8 (99.7; 99.9)	0.2	0.1	0.1
25–29	99.3 (99.0; 99.5)	0.8	0.5	0.6
30–34	97.3 (96.8; 97.8)	3.0	2.0	2.3
35–39	93.0 (92.2; 93.8)	7.9	4.3	4.8
40–44	86.7 (85.6; 87.8)	15.0	6.3	7.2
45–49	79.8 (78.4; 81.2)	22.8	6.9	7.8
50–54	75.1 (73.5; 76.7)	28.1	4.7	5.3
55–59	70.9 (69.0; 72.7)	32.9	4.2	4.7
60–64	68.7 (66.6; 70.7)	35.4	2.2	2.5
65	67.8 (65.4; 70.1)	36.4	0.9	1.0

*CI* Confidence Interval

*IPW* Inverse probability weighting

^*^Probability transformed to percentage

**Fig. 2:**
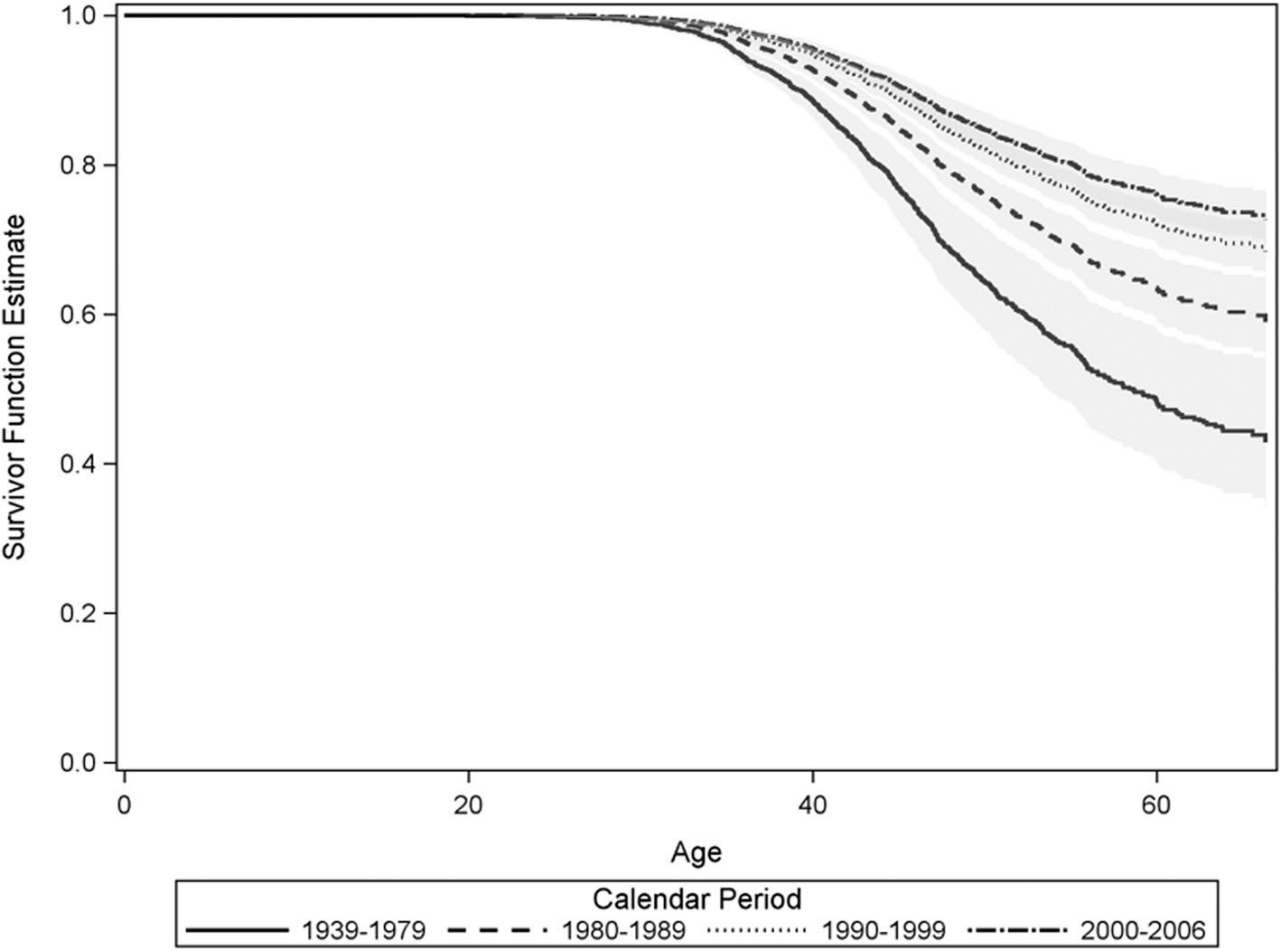
Simulated survival curves stratified by calendar period of hysterectomy, MARZY Hysterectomy Study

Figure 2 presents survivor functions stratified by the calendar periods when the hysterectomies were performed. Results show that women underwent hysterectomy at an increasingly older age in each successive calendar period between 1939 and 2006. The hazard ratios for the respective calendar periods, using 1939–1979 as reference, were: 1980–1989 (0.62, 95% CI 0.48–0.81), 1990–1999 (0.45, 95% CI 0.34–0.59), 2000–2006 (0.38, 95% CI 0.28–0.50). No violation of the proportionality assumption was found.

### Indications for hysterectomy

The indication for the hysterectomy was known for 664 women (69.1%). Among those, 61.4% reported a single indication for undergoing the procedure, while the remainder (38.6%) provided multiple.

Hysterectomies were primarily performed due to benign disease (86.7%, *n* = 576). The main indications were: 1) Leiomyoma of the uterus (46.1%, 95% CI 42.2–50.0%), 2) Abnormal uterine and vaginal bleeding (13.1%, 95% CI 10.6–15.9%), 3) Malignant, in situ and neoplasms of uncertain or unknown behaviour (12.2%, 95% CI 9.8–14.9%), 4) Genital prolapse (11.1%, 95% CI 8.9–13.8%), 5) Other non-inflammatory disorders of the uterus, except cervix (8.6%, 95% CI 6.6–11.0%) (Table 3).

**Table 3:** Main indication for hysterectomy (*n* = 664), MARZY Hysterectomy Study

Main indication for hysterectomy (ICD-10)	N	% (95 CI%)
Leiomyoma of uterus (D25)	306	46.1 (42.2 to 50.0)
Abnormal uterine bleeding (N92-N93)	87	13.1 (10.6 to 15.9)
Malignant or in situ cancer or neoplasms of uncertain and unknown behaviour (C00-C97, D00-D09, D37-D48)	81	12.2 (9.8 to 14.9)
Female genital prolapse (N81)	74	11.1 (8.9 to 13.8)
Other non-inflammatory disorders of uterus, except cervix (N85)	57	8.6 (6.6 to 11.0)
Pain and other conditions associated with female genital organs and menstrual cycle (N94)	12	1.8 (0.9 to 3.1)
Endometriosis (N80)	10	1.5 (0.7 to 2.8)
Benign neoplasm of ovary and other and unspecified female genital organs (D27-D28)	9	1.4 (0.6 to 2.6)
Maternal care for known or suspected abnormality of pelvic organs (O34)	6	0.9 (0.3 to 2.0)
Polyp of female genital tract (N84)	5	0.8 (0.2 to 1.7)
Dysplasia of cervix uteri (N87)	4	0.6 (0.2 to 1.5)
Other female pelvic inflammatory diseases (N73)	3	0.5 (0.1 to 1.3)
Contraceptive management (Z30)	3	0.5 (0.1 to 1.3)
Other	7	1.1 (0.4 to 2.2)
Total excluding missing^*^	**664**	**100.0**

^*^ Missing includes women for whom no indication for hysterectomy could be retrieved (*n* = 284) and information that could not be coded (^*n*^ = 13)

When analysing the indications over time, leiomyoma of the uterus consistently ranked first in all calendar periods (Table 4, Additional file 2).

**Table 4:** Main indication for hysterectomy according to calendar period (*n* = 651), MARZY Hysterectomy Study

Main indication for hysterectomy (ICD-10)	Period											
	**1939–1979**			**1980–1989**			**1990–1999**			**2000–2006**		
	**N**	**%**	**R**	**N**	**%**	**R**	**N**	**%**	**R**	**N**	**%**	**R**
Leiomyoma of uterus (D25)	30	40.0	1	83	46.6	1	114	47.5	1	73	46.2	1
Malignant or in situ cancer or neoplasms of uncertain or unknown behaviour (C00-C97, D00-D09, D37-D48)	14	18.7	2	21	11.8	3	21	8.8	5	24	15.2	2
Abnormal uterine and vaginal bleeding (N92-N93)	8	10.7	4	19	10.7	4	34	14.2	2	24	15.2	2
Female genital prolapse (N81)	11	14.7	3	22	12.4	2	29	12.1	3	11	7.0	3
Other non-inflammatory disorders of uterus, except cervix (N85)	8	10.7	4	15	8.4	5	24	10.0	4	9	5.7	4
Pain and other conditions associated with female genital organs and menstrual cycle (N94)	1	1.3	5	2	1.1	7	6	2.5	6	3	1.9	5
Endometriosis (N80)	1	1.3	5	3	1.7	6	4	1.7	7	2	1.3	6
Other	2	2.7	-	13	7.3	-	8	3.3	-	12	7.6	-
Total excluding missing indication and year^*^	**75**	**100.0**	**-**	**178**	**100.0**	**-**	**240**	**100.0**	**-**	**158**	**100.0**	**-**

*R* Rank

^*^Missing includes women for whom no indication/date for hysterectomy could be retrieved (*n* = 284), information that could not be coded (*n* = 13) or missing year (*n* = 13)

Indications for hysterectomy by age at hysterectomy (15–49 years and 50–65 years) are presented in the Additional file 3. Leiomyoma of the uterus was the most common reason for hysterectomy among both age groups (15–49: 49.0% and 50–65: 45.2%). Among those aged 50–65 years, female genital prolapse was the second most common reason for hysterectomy (13.0%), whereas fourth in younger women (4.7%). Contrastingly, abnormal uterine and vaginal bleeding accounted for 16.1% of the procedures in younger women, ranking second.

Finally, when examining the indications by calendar period (Additional files 4 and 5) among women aged 50–65 years at hysterectomy, leiomyoma of the uterus remained the most common in all periods, with no major shifts in indications over time.

## Discussion

In the recruitment sample of our population-based MARZY study, 20.4% of women aged 30–65 years reported having had a hysterectomy, with most procedures being conducted at ages 40–49 years. Benign disease was the main indication (86.8%) for hysterectomy in this population. Our simulations revealed a shift towards an older age at hysterectomy with an advancing calendar period, highlighting important changes in clinical practice in the last decades.

The prevalence of hysterectomy described in this study is in line with a nationally representative survey in Germany among women aged 18 to 79 years (17.5%) [14] as well as an analysis of six German population-based cohorts (18.7% to 23.7% in five of the six studies) [13]. Nevertheless, direct comparisons are difficult since the prevalence of hysterectomy is mainly affected by the age structure and characteristics of the studied populations [8, 13, 21].

In our study population, most women were hysterectomised between the ages of 40 and 49 (45.8%), with a peak at the ages 45 to 49 years (27.1%). Accordingly, the IPW-corrected age-specific probability of hysterectomy peaked at 45 to 49 years (7.8%), dropping thereafter. Similar patterns have been reported locally [14] and in other high-income countries, including Denmark [9], the United States [22] and Australia [8] and likely result from postponing hysterectomy to preserve fertility until women reach perimenopause and also the high frequency of gynaecological conditions in this age group [23–25].

The age-specific prevalence of hysterectomy in this study was highest (40.7%) among 60–64 year-olds. This reflects two aspects: the cumulative risk of hysterectomy as women age and the fact that the underlying clinical conditions of these women were managed under older clinical guidelines. The probability of undergoing a hysterectomy by 65 years was 36.4%, nearly twofold that reported in Australia (19.9%); the latter based on more recent data (2010–2012) [8].

Our simulated survival curves with partially non-overlapping confidence bands showed that women were hysterectomised at an increasingly older age in each successive calendar period. Over the past decades, there has been a trend towards uterine-sparing management of several gynaecological conditions, reducing the need for hysterectomy or enabling hysterectomies to be performed later in life [8]. The average age at the time of hysterectomy increased from 43.7 years in 1981 to 46.3 years in 2011 in Australia [8]. Although a continuous decrease in the hysterectomy rate has been observed in Germany, changing trends regarding age at hysterectomy had been thus far insufficiently reported [6, 13, 15].

Most hysterectomies were performed primarily due to benign disease, with leiomyoma accounting for about half of all procedures, regardless of age and calendar period. Abnormal uterine bleeding, cancer and genital prolapse had similar contributions (11–13%) to hysterectomy, with no clear changes over time, as opposed to reports from other countries [8–10, 22]. In Finland, genital prolapse has overtaken leiomyoma and was the most common indication in 2017–2018 [10]. In Australia, between 2010 and 2012, leiomyoma only ranked third after abnormal bleeding and genital prolapse [8]. In the United States, in 2010, leiomyoma and abnormal uterine bleeding were the two main reasons for hysterectomies with similar contributions [22]. The fact that we did not detect changes in the distribution of indications for hysterectomy over time could be due to our study design or the period captured by our analysis. The most important reductions in hysterectomy incidence in Germany were seen for leiomyoma due to changes in clinical management with increasing use of myomectomy in place of hysterectomy [2, 6, 14, 15]. Discrepancies in the distributions of indications for hysterectomies across countries and regions within countries reveal differences in disease burden in the population, clinical practice and inequalities in gynaecological care [10, 13, 14]. However, this can be minimized with the standardization of care via guidelines, as seen in Finland following the introduction of guidelines for surgical treatment of leiomyoma, abnormal bleeding, genital prolapse and endometriosis in 2005 [10].

The comparison between the age distribution of hysterectomised women at the time of the survey (peaking at 60–64 years) and the distribution of age at hysterectomy (peaking at 45–49 years) clearly shows the limitations of measuring the prevalence of hysterectomy at a single point in time. We addressed this constraint by conducting a time-to-event analysis to estimate the probability of undergoing a hysterectomy by age 65. By using the IPW method, we were able to estimate the probability of hysterectomy, including the women who did not provide the date when the procedure was performed (11.6%). This method can be used by future studies based on survey data to produce estimates beyond prevalence, even in the presence of missing information. Furthermore, we provided a detailed overview of the main indications for hysterectomy based on ICD-10, presenting them by calendar period and age group. Finally, our prevalence data can be used to correct incidence rates of gynaecological cancer (especially cervical and endometrial cancer) in Germany [26].

The main limitations were that our study population only included women between the ages 30–65 years and that data collection was cross-sectional, therefore reflecting a limited period. With regard to its cross-sectional design, it is possible that older women with less severe indications (i.e. non-malignant conditions) and comorbidities are somewhat overrepresented in our sample, considering that they were more likely to have survived until the time of the survey. Moreover, information regarding hysterectomy was self-reported and not independently verified by hospital records. However, hysterectomy is a major surgery that significantly impacts a woman's life; therefore, we consider this information reliable [27]. We acknowledge that there might be some imprecisions concerning the year of hysterectomy, especially for the hysterectomies performed several decades ago. Although we identified a consistently increasing age at hysterectomy with calendar period, no clear shifts in indication could be observed. Had we included more recent periods, it is possible that we would have detected further changes in the probability of hysterectomy and indications for it. Lastly, we did not collect information on the type of hysterectomy performed, which would have been useful to confirm shifts from more to less invasive procedures over time, as seen in other countries [9, 10, 22].

## Conclusions

One in five women in the population-based recruitment sample of the MARZY study had been hysterectomised and the risk of undergoing hysterectomy by age 65 years was 36.4%. The probability of hysterectomy changed over time: hysterectomies were performed at an older age with an advancing calendar period, underscoring changes in clinical practice likely supported by evolving national guidelines covering benign and malignant gynaecological diseases. Adherence to such guidelines might help achieve optimal and standardized care in Germany.

## Supplementary Information

**Additional file 1.** 

**Additional file 2.** 

**Additional file 3.** 

**Additional file 4.** 

**Additional file 5.**

## Data Availability

The datasets used and/or analysed during the current study are available from the corresponding author upon reasonable request.

## Abbreviations

CCS: Cervical cancer screening
HPV: Human papillomavirus
ICD: International Statistical Classification of Diseases and Related Health Problems
IPW: Inverse probability weighting
95% CI: 95% Confidence interval
