# Supplementary material for: Changes in the probability of hysterectomy in the city of Mainz and Mainz-Bingen region, Germany
Source: BMC Public Health. 2023 Jan 11;23:84. doi: 10.1186/s12889-022-14916-w (PMC9832650; doi:10.1186/s12889-022-14916-w)
Supplement: Supplementary file 1 — Additional file 1. [file 12889_2022_14916_MOESM1_ESM.docx]

# Additional File 1: Table – Sociodemographic characteristics of women who reported undergoing a hysterectomy (n= 961), MARZY Hysterectomy Study

|  | **N** | **%** |
| --- | --- | --- |
| **Place of residence** |  |  |
| Mainz-Bingen district | 554 | 57.6 |
| City of Mainz | 407 | 42.4 |
| **Nationality** |  |  |
| German | 631 | 65.7 |
| Other | 74 | 7.7 |
| Missing | 256 | 26.6 |
| **Total** | **961** | **100.0** |
