# Supplementary material for: Changes in the probability of hysterectomy in the city of Mainz and Mainz-Bingen region, Germany
Source: BMC Public Health. 2023 Jan 11;23:84. doi: 10.1186/s12889-022-14916-w (PMC9832650; doi:10.1186/s12889-022-14916-w)
Supplement: Supplementary file 3 — Additional file 3. [file 12889_2022_14916_MOESM3_ESM.docx]

# Additional File 3: Table – Main indication for hysterectomy according to age at hysterectomy (n= 664), MARZY Hysterectomy Study

| **Main indication for hysterectomy (ICD-10)** | **Age group** | | | | | |
| --- | --- | --- | --- | --- | --- | --- |
|  | **15-49 years** | | | **50-65 years** | | |
|  | **N** | **%** | **R** | **N** | **%** | **R** |
| Leiomyoma of uterus (D25) | 73 | 49.0 | 1 | 233 | 45.2 | 1 |
| Female genital prolapse (N81) | 7 | 4.7 | 4 | 67 | 13.0 | 2 |
| Abnormal uterine and vaginal bleeding (N92-N93) | 24 | 16.1 | 2 | 63 | 12.2 | 3 |
| Malignant or in situ cancer or neoplasms of uncertain or unknown behaviour (C00-C97, D00-D09, D37-D48) | 23 | 15.4 | 3 | 58 | 11.3 | 4 |
| Other non-inflammatory disorders of uterus, except cervix (N85) | 6 | 4.0 | 5 | 51 | 9.9 | 5 |
| Other | 16 | 10.7 | - | 43 | 8.3 | - |
| **Total excluding missing indication^*^** | **149** | **100.0** | **-** | **515** | **100.0** | **-** |

R = Rank

_*_ Missing includes women for whom no indication/date for hysterectomy could be retrieved (n= 284) and information that could not be coded
(n= 13)
