# Supplementary material for: Changes in the probability of hysterectomy in the city of Mainz and Mainz-Bingen region, Germany
Source: BMC Public Health. 2023 Jan 11;23:84. doi: 10.1186/s12889-022-14916-w (PMC9832650; doi:10.1186/s12889-022-14916-w)
Supplement: Supplementary file 4 — Additional file 4. [file 12889_2022_14916_MOESM4_ESM.docx]

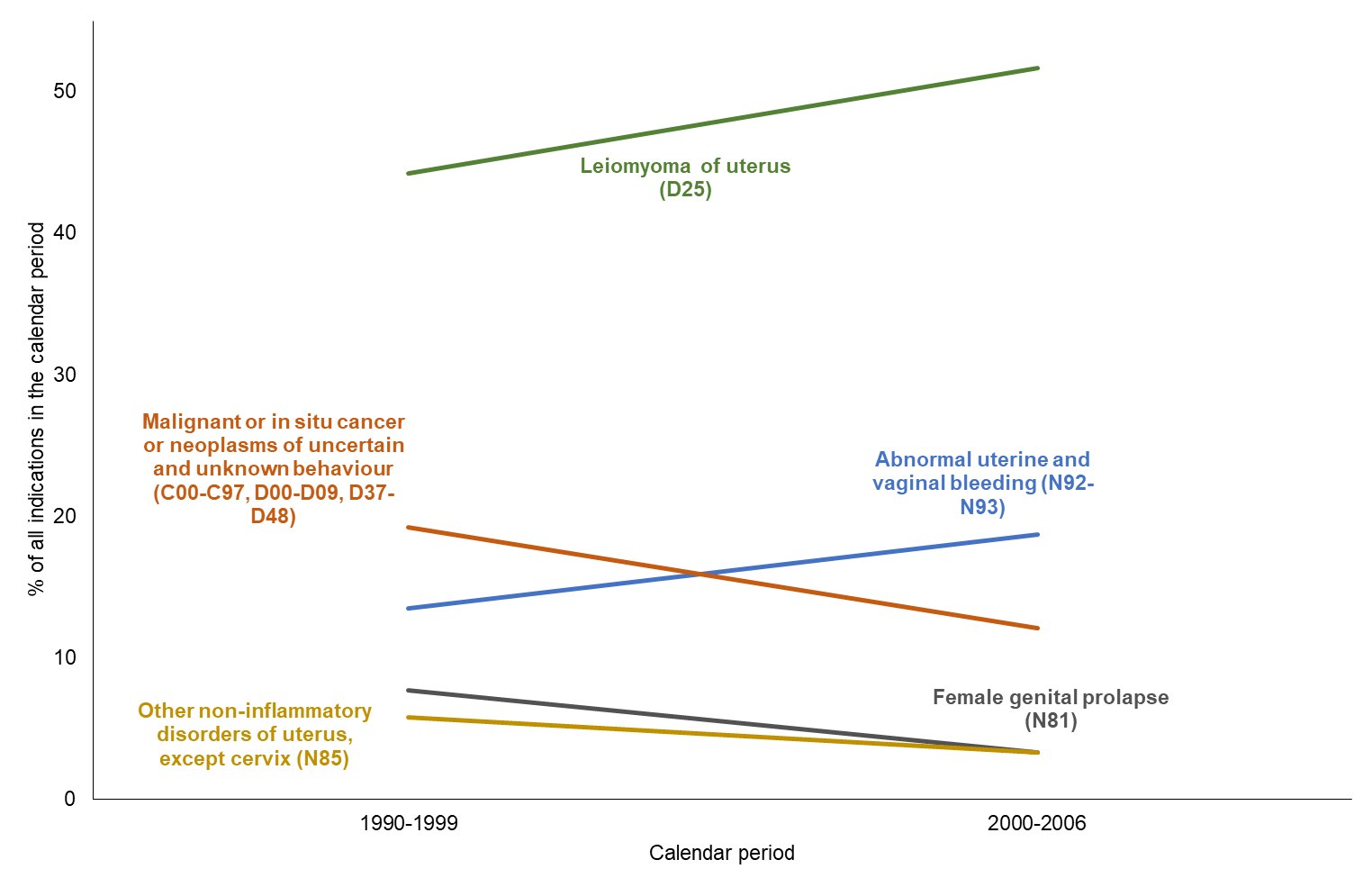


# Additional File 4: Figure – Most common indications for hysterectomy by calendar period (1990-1999, 2000-2006) among women aged 15-49 years at hysterectomy, MARZY Hysterectomy Study
